# Adjuvant chemotherapy and survival among patients 70 years of age and younger with node-negative breast cancer and the 21-gene recurrence score of 26–30

**DOI:** 10.1186/s13058-019-1190-4

**Published:** 2019-10-16

**Authors:** Seho Park, Yunan Han, Ying Liu, Adetunji T. Toriola, Lindsay L. Peterson, Graham A. Colditz, Seung Il Kim, Young Up Cho, Byeong-Woo Park, Yikyung Park

**Affiliations:** 1 Division of Public Health Sciences, Department of Surgery, Washington University School of Medicine, 660 South Euclid Avenue, Campus Box 8100, St. Louis, MO 63110 USA; 2 Division of Breast Surgery, Department of Surgery, Yonsei University College of Medicine, Seoul, Republic of Korea; 3 Department of Breast Surgery, First Hospital of China Medical University, Shenyang, China; 4 Division of Medical Oncology, Department of Medicine, Washington University School of Medicine, St. Louis, MO USA

**Keywords:** Breast cancer, Chemotherapy, Survival, 21-gene recurrence score

## Abstract

**Background:**

The benefits of chemotherapy in node-negative, hormone receptor-positive, and human epidermal growth factor receptor 2 (HER2)-negative breast cancer patients with the 21-gene recurrence score (RS) of 18–30, particularly those with RS 26–30, are not known.

**Methods:**

Using the Surveillance, Epidemiology, and End Results (SEER) data, we retrospectively identified 29,137 breast cancer patients with the 21-gene RS of 18–30 diagnosed between 2004 and 2015. Mortality risks according to the RS and chemotherapy use were compared by the Kaplan-Meier method and Cox’s proportional hazards model.

**Results:**

Among the breast cancer patients with the RS 18–30, 21% of them had RS 26–30. Compared to breast cancer patients with RS 18–25, patients with RS 26–30 had more aggressive tumor characteristics and chemotherapy use and increased risk of breast cancer-specific mortality and overall mortality. In breast cancer patients who were aged ≤ 70 years and had RS of 26–30, chemotherapy administration was associated with a 32% lower risk of breast cancer-specific mortality (hazard ratio [HR], 0.68; 95% confidence interval [CI], 0.47–0.99) and a 42% lower risk of overall mortality (HR, 0.58; 95% CI, 0.44–0.76). Survival benefits were most pronounced in breast cancer patients who were younger or had grade III tumor.

**Conclusions:**

The 21-gene RS of 18–30 showed heterogeneous outcomes, and the RS 26–30 was a significant prognostic factor for an increased risk of mortality. Adjuvant chemotherapy could improve the survival of node-negative, hormone receptor-positive, and HER2-negative breast cancer patients with the 21-gene RS 26–30 and should be considered for patients, especially younger patients or patients with high-grade tumors.

**Electronic supplementary material:**

The online version of this article (10.1186/s13058-019-1190-4) contains supplementary material, which is available to authorized users.

## Introduction

Systemic chemotherapy reduces the risk of recurrence and mortality in estrogen receptor (ER) and/or progesterone receptor (PR)-positive breast cancer patients irrespective of lymph node status [1]. However, the benefits of chemotherapy are not experienced by all breast cancer patients, leading to a need to identify patients who are more likely to benefit from adjuvant chemotherapy. The 21-gene recurrence score (RS) assay (Oncotype DX®; Genomic Health, Inc., Redwood City, CA) available since 2004 is one of the promising tools to guide treatment decisions in hormone receptor-positive and human epidermal growth factor receptor 2 (HER2)-negative breast cancer patients [2, 3]. Based on the 21-gene RS assay, patients are classified into high (score ≥ 31), intermediate (score 18–30), and low (score ≤ 17) risk. Studies found that patients in the high-risk group of RS ≥ 31 had a significant benefit from chemotherapy [4], while patients with the RS < 11 or 11–25 who were treated with endocrine therapy alone had excellent survival outcomes without chemotherapy in the Trial Assigning Individualized Options for Treatment (TAILORx) trial [5, 6].

Although patients with the intermediate 21-gene RS of 18–30 seemed to have no clinical benefits from chemotherapy in previous studies [3, 7, 8], young women of ≤ 50 years old with the RS 16–25 who received chemotherapy had lower distant recurrence rates than women who did not receive chemotherapy [6]. A recent study using the National Cancer Data Base (NCDB) also found that combination of chemotherapy and hormone therapy was associated with a lower risk of overall mortality in node-negative breast cancer patients with the RS 18–25 as well as those with the RS 26–30 than patients who did not receive chemotherapy [9]. However, this study included older patients (> 70 years of age) and those with very small tumors of ≤ 0.5 cm, or favorable histologies, for whom the National Comprehensive Cancer Network (NCCN) clinical practice guidelines for breast cancer do not recommend multigene assays [10]; therefore, more specified analyses focusing on an intermediate RS group are required.

Given that the uncertainty of chemotherapy benefits remains in intermediate-risk patients, especially with the RS of 26–30 in hormone receptor-positive, HER2-negative, and node-negative breast cancers, we investigated whether adjuvant chemotherapy was associated with breast cancer-specific mortality and overall mortality in breast cancer patients with the RS 18–30, particularly among those aged ≤ 70 years with the RS 26–30.

## Patients and methods

### The Surveillance, Epidemiology, and End Results (SEER) database

Since 1973, the SEER has collected cancer incidence and survival data, including patient demographics; tumor characteristics of primary site, grade, and stage at diagnosis; and first course of treatments from 18 population-based cancer registries. The SEER registry has also collected ER and PR status of breast cancer since 1990 and HER2 status since 2010. All breast cancers diagnosed between 2004 and 2015 (excluding cases reported by the Alaska Native Tumor Registry) were linked to the 21-gene RS data provided by the Genomic Health Clinical Laboratory [11]. To maximize the use of Oncotype DX data and have longer follow-up time, we included all 21-gene RS data since its first collection in 2004. We obtained permission to use the SEER custom data with additional radiation therapy and chemotherapy information and the 21-gene RS data.

### Study population

We identified 111,635 breast cancer cases with the 21-gene RS in the SEER 18 database from 2004 to 2015. We first excluded male breast cancer patients and cases identified at death/autopsy or through death certificate only (Additional file 1: Figure S1). Based on the NCCN guidelines for use of multigene assay [10], we also excluded cases with at least one of the following conditions: tumors with non-epithelial origin including sarcoma (International Classification of Diseases for Oncology, 3rd Edition, ICD-O-3 > 8800), tubular (8211), mucinous (8453, 8480, and 8481), and papillary (8050, 8260, and 8503) subtypes; T0-T1a or T4 stage; lymph node-positive (≥ N1mi); distant metastasis (M1) or unknown stage at diagnosis; ER/PR-negative or unknown ER/PR status; or cases whose 21-gene RS results were obtained > 12 months after breast cancer diagnosis. In addition, we excluded breast cancer cases with the 21-gene RS < 18 (low-risk category) or > 30 (high-risk category). Because HER2 status was available from 2010, we divided the study population into two groups based on time, 2004–2009 and 2010–2015. In the 2010–2015 dataset, we further excluded breast cancer cases whose HER2 status was positive, borderline, or unknown. The final analytic cohorts consisted of 9346 breast cancer patients in 2004–2009 dataset and 19,791 patients in 2010–2015 dataset. Because of our de-identified data release from the SEER program and study completion in accordance with the SEER data-use agreement, this study was exempted from the Institutional Review Board approval.

### Clinicopathological information and outcomes

The SEER data provided patient’s demographics, such as age at cancer diagnosis; race/ethnicity; marital status; history of other cancer; tumor characteristics including morphology, stage, grade, and ER/PR status; and first course of treatment—type of surgery (breast-conservation surgery and mastectomy), radiation therapy (yes and no/unknown), and chemotherapy (yes and no/unknown). We defined breast cancer histology: ductal (ICD-O-3, 8500), lobular (8520), mixed ductal-lobular (8522, 8523, and 8524), and other type.

Underlying causes of death and durations of survival in the SEER registries were ascertained through linkage to the state death certificates and the National Death Index from the National Center for Health Statistics [12]. We used these variables recorded in the SEER database for calculating breast cancer-specific survival and overall survival.

### Statistical analysis

We conducted analyses in the 2004–2009 and 2010–2015 (HER2-negative case only) dataset, separately, and the 2004–2015 combined datasets. Based on cutoffs in the TAILORx trial [13], we categorized patients into the 21-gene RS 18–25 and 26–30 groups and compared demographic and clinicopathological characteristics: differences in means for continuous variables and percentages for categorical variables between two groups were tested using *t* test and chi-square test, respectively. Survival curves were plotted by the Kaplan-Meier method and differences in survival time were calculated by the log-rank test.

Person-months of follow-up were calculated from the date of breast cancer diagnosis to date of death, known last follow-up, or follow-up end date (December 31, 2015), whichever occurred first. Hazard ratios (HRs) and 95% confidence intervals (CIs) for breast cancer-specific and all-cause mortality were estimated by the Cox’s proportional hazards model with person-months as time scale. A missing category was created for race/ethnicity (0.5%), marital status (4.2%), and grade (2.3%). We examined age-adjusted and multivariate models adjusting for age at diagnosis, calendar year of breast cancer diagnosis (2-year interval), race/ethnicity (White, Black, and others), marital status (married and single/other), history of cancer (yes and no), histologic type (ductal, lobular, and mixed ductal-lobular/other), tumor stage (T1b, T1c, and T2–3), grade (I, II, and III), and ER/PR status (ER+/PR+, ER+/PR−, and ER−/PR+), surgery (breast-conservation surgery and mastectomy), radiation therapy (yes, and no/unknown), and chemotherapy (yes and no/unknown). We also calculated the mortality risk by chemotherapy status in patients who had the 21-gene RS 26–30 and were ≤ 70 years old. We conducted sensitivity analyses in the 2010–2015 dataset by including breast cancer cases with HER2 positive, borderline, or unknown.

Breast cancer cases were generated and exported using SEER*Stat software version 8.3.5 (https://seer.cancer.gov/seerstat). All statistical analyses were performed using SAS software version 9.4 (SAS Institute Inc., Cary, NC), and a *P* value < 0.05 was considered statistically significant for 2-sided tests.

## Results

### Mortality risk according to the 21-gene RS

There were 19,791 breast cancer patients in the 2010–2015 (mean follow-up 32 months) and 9346 patients in the 2004–2009 dataset (mean follow-up 89 months; Table 1). In both datasets, 21% of patients had the 21-gene RS of 26–30. The distribution of the 21-gene RS was not different between the 2010–2015 and 2004–2009 datasets (Additional file 2: Figure S2). Compared to patients with the RS 18–25, those with the 21-gene RS 26–30 were more likely to be older, non-White, not married, have ductal histology, advanced tumor stage, higher grade tumor, or frequent loss of ER or PR, and receive chemotherapy, but no/unknown radiation therapy.

**Table 1:** Demographic and clinicopathological characteristics of study population

	2010–2015 dataset (*n* = 19,791)			2004–2009 dataset (*n* = 9346)		
	RS 18–25 [*n* = 15,731; No. (%)]	RS 26–30 [*n* = 4060; No. (%)]	*P*	RS 18–25 [*n* = 7401; No. (%)]	RS 26–30 [*n* = 1945; No. (%)]	*P*
Year of diagnosis						
2004–2005	–	–		567 (7.7)	155 (8.0)	0.421
2006–2007	–	–		2522 (34.1)	689 (35.4)	
2008–2009	–	–		4312 (58.3)	1101 (56.6)	
2010–2011	4748 (30.2)	1243 (30.6)	0.797	–	–	
2012–2013	5229 (33.2)	1329 (32.7)		–	–	
2014–2015	5754 (36.6)	1488 (36.7)		–	–	
Age at diagnosis (years)						
≤ 50	3836 (24.4)	923 (22.7)	0.025	2340 (31.6)	539 (27.7)	0.003
51–60	4924 (31.3)	1239 (30.5)		2456 (33.2)	654 (33.6)	
61–70	4990 (31.7)	1378 (33.9)		1971 (26.6)	557 (28.6)	
> 70	1981 (12.6)	520 (12.8)		634 (8.6)	195 (10.0)	
Race/ethnicity						
White	12,946 (82.3)	3263 (80.4)	0.018	6299 (85.1)	1662 (85.5)	0.737
Black	1296 (8.2)	391 (9.6)		506 (6.8)	139 (7.2)	
Other*	1405 (8.9)	386 (9.5)		561 (7.6)	137 (7.0)	
Missing	84 (0.5)	20 (0.5)		35 (0.5)	7 (0.4)	
History of cancer						
No	13,960 (88.7)	3551 (87.5)	0.023	6654 (89.9)	1764 (90.7)	0.302
Yes	1771 (11.3)	509 (12.5)		747 (10.1)	181 (9.3)	
Marital status						
Married	9819 (62.4)	2473 (60.9)	0.004	4924 (66.5)	1259 (64.7)	0.215
Single	2105 (13.4)	630 (15.5)		885 (12.0)	255 (13.1)	
Other^†^	3078 (19.6)	786 (19.4)		1333 (18.0)	372 (19.1)	
Missing	729 (4.6)	171 (4.2)		259 (3.5)	59 (3.0)	
Histologic type						
Ductal	11,490 (73.0)	3226 (79.5)	< 0.001	5446 (73.6)	1556 (80.0)	< 0.001
Lobular	2251 (14.3)	391 (9.6)		897 (12.1)	161 (8.3)	
Mixed ductal-lobular	1818 (11.6)	394 (9.7)		960 (13.0)	202 (10.4)	
Other	172 (1.1)	49 (1.2)		98 (1.3)	26 (1.3)	
Tumor stage						
T1b	3535 (22.5)	801 (19.7)	< 0.001	1789 (24.2)	409 (21.0)	< 0.001
T1c	8271 (52.6)	2112 (52.0)		4217 (57.0)	1070 (55.0)	
T2	3698 (23.5)	1091 (26.9)		1339 (18.1)	446 (22.9)	
T3	227 (1.4)	56 (1.4)		56 (0.8)	20 (1.0)	
Grade						
I	3774 (24.0)	486 (12.0)	< 0.001	1795 (24.3)	251 (12.9)	< 0.001
II	8990 (57.2)	2087 (51.4)		4145 (56.0)	1007 (51.8)	
III	2644 (16.8)	1434 (35.3)		1233 (16.7)	636 (32.7)	
Missing	323 (2.1)	53 (1.3)		228 (3.1)	51 (2.6)	
ER/PR status						
+/+	13,842 (88.0)	3164 (77.9)	< 0.001	6389 (86.3)	1459 (75.0)	< 0.001
+/−	1872 (11.9)	885 (21.8)		993 (13.4)	478 (24.6)	
−/+	17 (0.1)	11 (0.3)		19 (0.3)	8 (0.4)	
Type of surgery						
Breast-conservation surgery	10,806 (68.7)	2747 (67.7)	0.207	5199 (70.3)	1326 (68.2)	0.076
Mastectomy	4925 (31.3)	1313 (32.3)		2202 (29.8)	619 (31.8)	
Radiation therapy						
No or unknown	6268 (39.8)	1834 (45.2)	< 0.001	3038 (41.1)	853 (43.9)	0.025
Yes	9463 (60.2)	2226 (54.8)		4363 (59.0)	1092 (56.1)	
Chemotherapy						
No or unknown	11,832 (75.2)	1807 (44.5)	< 0.001	5044 (68.2)	865 (44.5)	< 0.001
Yes	3899 (24.8)	2253 (55.5)		2357 (31.9)	1080 (55.5)	

*RS* recurrence score, *No*. number, *ER* estrogen receptor, *PR* progesterone receptor

*Other race includes American Indian, Alaska Native, and Asian or Pacific Islander

^†^Other marital status includes separated, divorced, and widowed categories

**Fig. 1:**
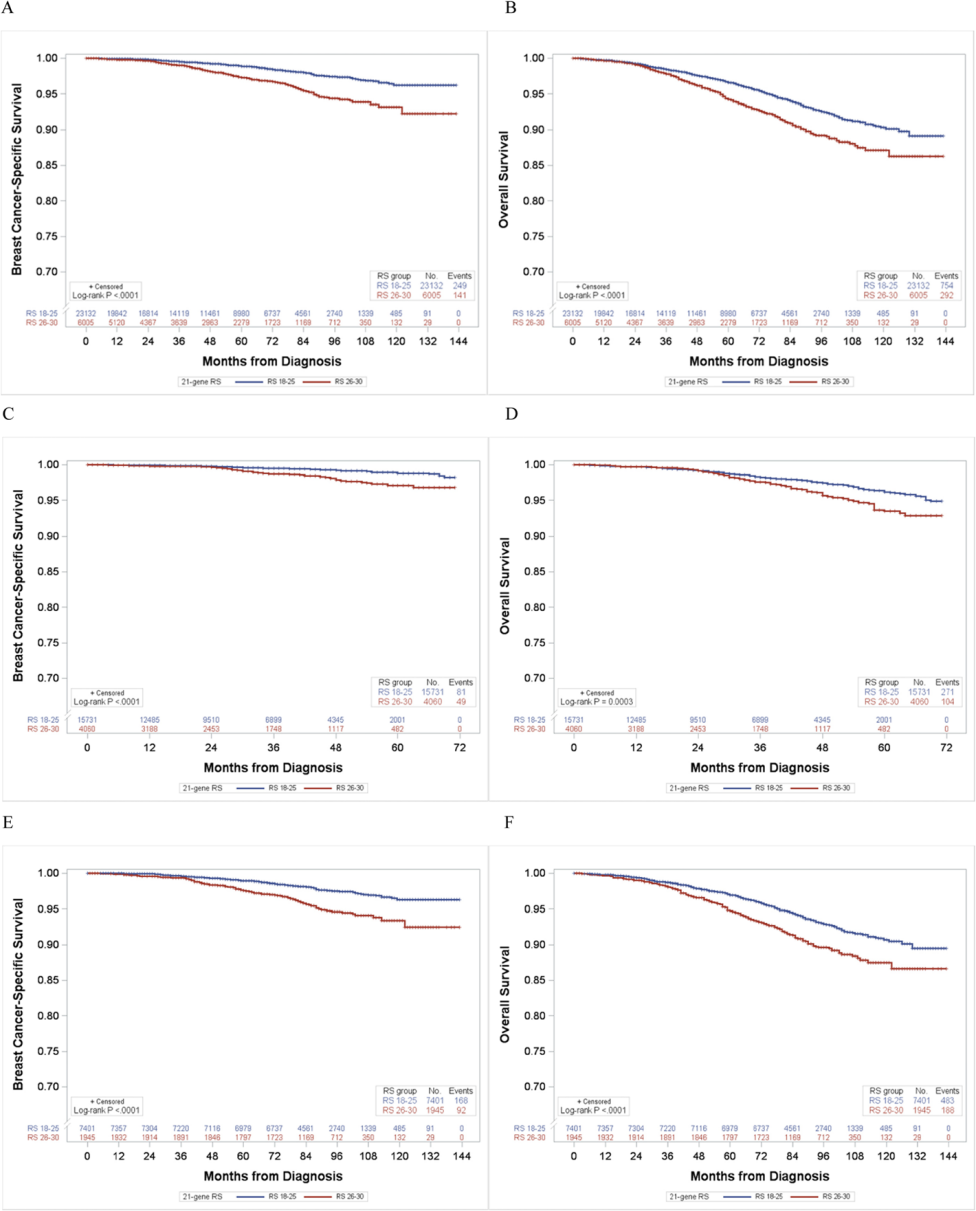
Breast cancer-specific and overall survival curves according to the 21-gene recurrence score (RS). **a** Breast cancer-specific survival and **b** overall survival in 2004–2015 (*n* = 29,137). **c** Breast cancer-specific survival and **d** overall survival in 2010–2015 (*n* = 19,791). **e** Breast cancer-specific survival and **f** overall survival in 2004–2009 (*n* = 9346)

Patients with the RS 26–30 in the 2004–2015 dataset had significantly worse breast cancer-specific survival and overall survival compared to those with the RS 18–25 (Fig. 1a, b). When the survival was examined in the 2010–2015 and 2004–2009 dataset, separately, we found similar results: poor survival in women with the RS 26–30 in each dataset (Fig. 1c–f). Compared to patients with the RS 18–25, those with the RS 26–30 had an 81% increased risk of breast cancer-specific mortality (HR, 1.81; 95% CI, 1.46–2.26) and a 37% increased risk of overall mortality (HR,1.37; 95% CI, 1.19–1.58; Table 2) after adjusting for potential confounders, including age at diagnosis, clinicopathological characteristics, and treatments. Similar results were consistently observed when we analyzed the 2010–2015 and 2004–2009 dataset, separately.

**Table 2:** Hazard ratios (HR) for breast cancer-specific and overall mortality by the 21-gene RS

	Breast cancer-specific mortality		Overall mortality	
	RS 18–25 (Reference)	RS 26–30 [HR (95% CI)]	RS 18–25 (Reference)	RS 26–30 [HR (95% CI)]
2004–2015 dataset (*n* = 29,137)				
No. of events	249	141	754	292
Age-adjusted model	1.00	2.17 (1.76 to 2.67)	1.00	1.43 (1.25 to 1.64)
Age- and clinicopathological factor-adjusted model	1.00	1.81 (1.46 to 2.24)	1.00	1.32 (1.15 to 1.52)
Age- and clinicopathological and treatment factor-adjusted model	1.00	1.81 (1.46 to 2.26)	1.00	1.37 (1.19 to 1.58)
Continuous RS in age- and clinicopathological and treatment factor-adjusted model*	1.09 (1.06 to 1.12)/1-unit RS		1.05 (1.03 to 1.07)/1-unit RS	
2010–2015 dataset (*n* = 19,791)				
No. of events	81	49	271	104
Age-adjusted model	1.00	2.35 (1.65 to 3.36)	1.00	1.46 (1.16 to 1.83)
Age- and clinicopathological factor-adjusted model	1.00	1.84 (1.27 to 2.67)	1.00	1.27 (1.01 to 1.61)
Age- and clinicopathological and treatment factor-adjusted model	1.00	1.83 (1.25 to 2.69)	1.00	1.28 (1.00 to 1.63)
Continuous RS in age- and clinicopathological and treatment factor-adjusted model*	1.12 (1.06 to 1.18)/1-unit RS		1.05 (1.02 to 1.08)/1-unit RS	
2004–2009 dataset (*n* = 9346)				
No. of events	168	92	483	188
Age-adjusted model	1.00	2.08 (1.61 to 2.68)	1.00	1.42 (1.20 to 1.68)
Age- and clinicopathological factor-adjusted model	1.00	1.79 (1.38 to 2.33)	1.00	1.35 (1.14 to 1.61)
Age- and clinicopathological and treatment factor-adjusted model	1.00	1.80 (1.38 to 2.36)	1.00	1.41 (1.18 to 1.69)
Continuous RS in age- and clinicopathological and treatment factor-adjusted model*	1.07 (1.04 to 1.11)/1-unit RS		1.05 (1.03 to 1.07)/1-unit RS	

Age at diagnosis is used by categorization into ≤ 50, 51–60, 61–70, and > 70 years. Adjusted clinicopathological factors are year of diagnosis (2004–2005, 2006–2007, 2008–2009 in 2004–2009 dataset and 2010–2011, 2012–2013, and 2014–2015 in 2010–2015 dataset), race/ethnicity (White, Black, and other), history of cancer (no and yes), marital status (married and single/other), histologic type (ductal, lobular, and mixed ductal-lobular/other), tumor stage (T1b, T1c, and T2–3), grade (I, II, III, and missing), and ER/PR status (both ER/PR-positive and either ER/PR-positive). Treatment factors are type of surgery (breast-conservation surgery and mastectomy), radiation therapy (no/unknown and yes), and chemotherapy (no/unknown and yes)

*RS* recurrence score, *HR* hazard ratio, *CI* confidence interval, *No.* number

*The 21-gene RS is entered as a continuous variable into Cox’s hazard models adjusting for age at diagnosis and clinicopathological and treatment factors. HR per 1 unit increase in RS

**Fig. 2:**
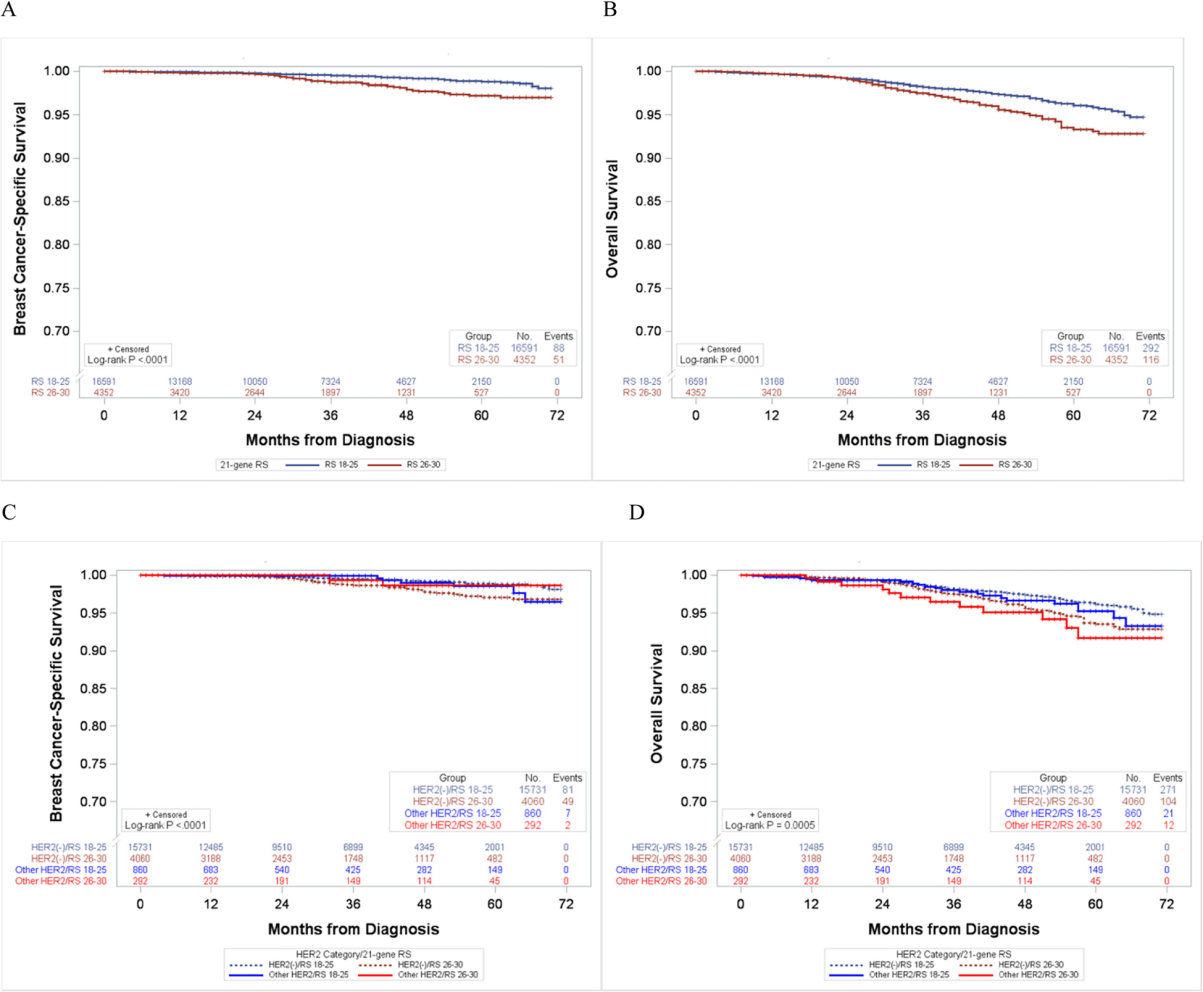
Breast cancer-specific and overall survival curves according to the 21-gene RS in sensitivity analyses that included breast cancer with HER2-positive, borderline, and unknown status in 2010–2015 (*n* = 20,943). **a** Breast cancer-specific survival and **b** overall survival according to RS groups. **c** Breast cancer-specific survival and **d** overall survival according to RS group by HER2 status

In sensitivity analyses that included breast cancer with HER2-positive, borderline, or unknown status in 2010–2015 dataset (*n* = 1152, 5.5% of total breast cancer cases), similar results were found: patients with the RS 26–30 had poor survival than those with the RS 18–25 (Fig. 2). Also, after further adjusting for HER2 status in multivariate models, there was a 78% and 31% increased risk of breast cancer-specific and overall mortality, respectively, in patients with the RS 26–30 compared to those with the RS 18–25 (Additional file 3; Table S1).

### Chemotherapy and survival of patients with the RS 26–30 and age ≤ 70 years

**Fig. 3:**
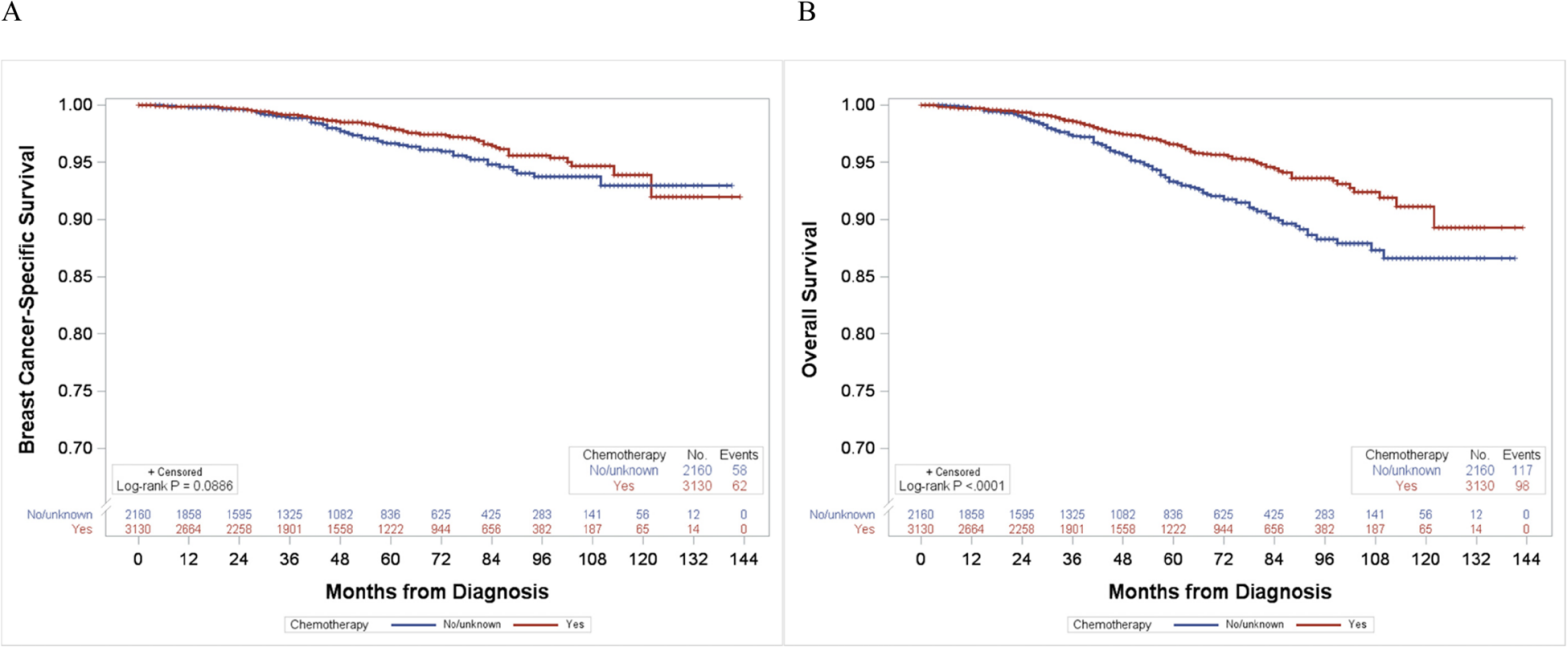
Breast cancer-specific and overall survival curves according to chemotherapy use in patients aged ≤ 70 years old with the RS 26–30 in the SEER 2004–2015 dataset (*n* = 5290). **a** Breast cancer-specific survival and **b** overall survival

**Fig. 4:**
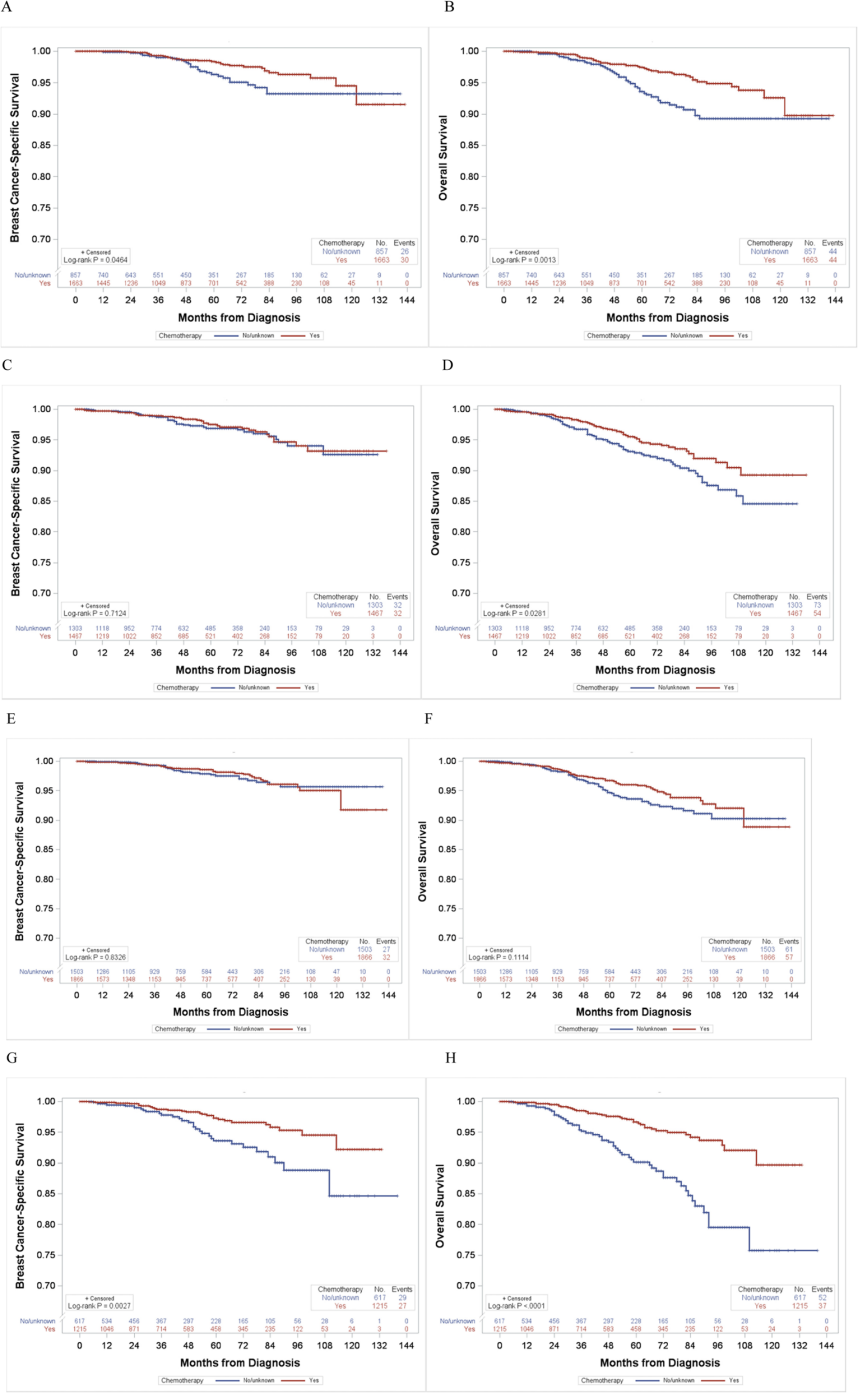
Breast cancer-specific and overall survival curves according to chemotherapy use among patients with age ≤ 70 years and the RS 26–30, stratified by age and tumor grade. **a** Breast cancer-specific survival in patients ≤ 56 years of age, **b** overall survival in patients ≤ 56 years of age, **c** breast cancer-specific survival in patients 57–70 years of age, **d** overall survival in patients 57–70 years of age, **e** breast cancer-specific survival in patients with grade I–II tumor, **f** overall survival in patients with grade I–II tumor, **g** breast cancer-specific survival in patients with grade III tumor, and **h** overall survival in patients with grade III tumor

Among 5290 breast cancer patients aged ≤ 70 years with the RS 26–30, 3130 (59%) patients received adjuvant chemotherapy. Patients who received chemotherapy were more likely to be younger and have advanced tumor stage, grade III, and also receiving radiation therapy than those who did not (Additional file 4: Table S2). In the 2004–2015 dataset, breast cancer-specific survival did not significantly differ by chemotherapy use (log-rank *P* = 0.089; Fig. 3a). On the other hand, overall survival rate was significantly higher in patients who received chemotherapy than in those who did not (log-rank *P* < 0.001; Fig. 3b). When breast cancer-specific survival by chemotherapy use was examined in subgroups of age (≤ 56 and 57–70 years old: mean age = 56 years) and grade (I–II and III), younger patients of ≤ 56 years (log-rank *P* = 0.046; Fig. 4a) and patients with grade III tumor (log-rank *P* = 0.003; Fig. 4g) tended to have better survival when they received chemotherapy than those who did not. Overall survival was consistently higher in patients who received chemotherapy regardless of their age and grade (Fig. 4). Similar results were found in analyses using the 2004–2009 and 2010–2015 data, separately (data not shown).

In multivariate Cox’s hazard models, adjuvant chemotherapy was associated with significantly decreased risks of breast cancer-specific mortality (HR, 0.68; 95% CI, 0.47–0.99) and overall mortality (HR, 0.58; 95% CI, 0.44–0.76) after adjusting for demographic, clinicopathological, and treatment factors in the 2004–2015 dataset (Table 3). Similar, but statistically not significant, associations were observed in analyses of the 2010–2015 and 2004–2009 datasets, separately.

**Table 3:** Hazard ratios (HR) for breast cancer-specific and overall mortality by chemotherapy use in patients aged ≤ 70 years old with the RS 26–30

	Breast cancer-specific mortality		Overall mortality	
	No/unknown chemotherapy (Reference)	Yes chemotherapy [HR (95% CI)]	No/unknown chemotherapy (Reference)	Yes chemotherapy [HR (95% CI)]
2004–2015 dataset (*n* = 5290)				
No. of events	58	62	117	98
Age-adjusted model	1.00	0.76 (0.53 to 1.09)	1.00	0.63 (0.48 to 0.83)
Age- and clinicopathological factor-adjusted model	1.00	0.66 (0.45 to 0.95)	1.00	0.56 (0.42 to 0.74)
Age- and clinicopathological and treatment factor-adjusted model	1.00	0.68 (0.47 to 0.99)	1.00	0.58 (0.44 to 0.76)
2010–2015 dataset (*n* = 3540)				
No. of events	22	23	40	40
Age-adjusted model	1.00	0.73 (0.40 to 1.32)	1.00	0.75 (0.48 to 1.18)
Age- and clinicopathological factor-adjusted model	1.00	0.63 (0.34 to 1.15)	1.00	0.67 (0.43 to 1.05)
Age- and clinicopathological and treatment factor-adjusted model	1.00	0.64 (0.35 to 1.18)	1.00	0.72 (0.45 to 1.14)
2004–2009 dataset (*n* = 1750)				
No. of events	36	39	77	58
Age-adjusted model	1.00	0.77 (0.49 to 1.23)	1.00	0.56 (0.40 to 0.79)
Age- and clinicopathological factor-adjusted model	1.00	0.68 (0.43 to 1.09)	1.00	0.50 (0.35 to 0.71)
Age- and clinicopathological and treatment factor-adjusted model	1.00	0.71 (0.44 to 1.13)	1.00	0.51 (0.36 to 0.72)

Age at diagnosis is used by categorization into ≤ 50, 51–60, and 61–70 years. Adjusted clinicopathological factors are year of diagnosis (2004–2005, 2006–2007, 2008–2009 in 2004–2009 dataset and 2010–2011, 2012–2013, and 2014–2015 in 2010–2015 dataset), race/ethnicity (White, Black, and other), history of cancer (no and yes), marital status (married and single/other), histologic type (ductal, lobular, and mixed ductal-lobular/other), tumor stage (T1b, T1c, and T2–3), grade (I, II, III, and missing), and ER/PR status (both ER/PR-positive and either ER/PR-positive). Treatment factors are type of surgery (breast-conservation surgery and mastectomy) and radiation therapy (no/unknown and yes)

*RS* recurrence score, *HR* hazard ratio, *CI* confidence interval, *No*. number

## Discussion

In this study, we found that the 21-gene RS was associated with an increased risk of breast cancer-specific and overall mortality in breast cancer patients who had the RS18–30. In addition, our study suggested that adjuvant chemotherapy was associated with better survival of patients aged ≤ 70 years with the 21-gene RS 26–30. Interestingly, we found that breast cancer-specific and overall survival rates in patients with the RS 26–30 and who received chemotherapy were higher in younger versus older patients and those with grade III versus grade I–II tumor. Additional clinical parameters such as Ki-67 proliferative index or expression levels of hormone receptor, which represent tumor biology or subtype, may provide additional information to aid a treatment decision for this subgroup of breast cancer patients [14, 15].

Since the introduction of multigene assays, use of the 21-gene RS test in clinical practice has gradually increased and is anticipated to be widely utilized as a treatment decision aid in ER-positive and HER2-negative early-stage breast cancer patients [16, 17]. However, clinicians should be aware that the 21-gene RS is a relative numeric calculated, not an absolute parameter for predicting patient prognosis. In previous clinical trials that validated the 21-gene RS assay [2, 3], the prevalence of patients with the RS 18–30 classified as an intermediate-risk group was 21–22%. However, the prevalence of breast cancer with the RS 18–30 was higher (33%) in a nation-wide cancer database in the USA [9]. The prevalence of the intermediate RS group varied across countries: from 20.0% in the Japanese to 40.7% in the Israeli with early-stage breast cancer [7, 18, 19]. Among breast cancer patients with intermediate risk of the RS 18–30, approximately one out of five patients had the RS of 26–30 in our study using the SEER and the previous studies [7, 9].

Similarly to previous studies [7, 9], we observed that the intermediate-risk group, RS 18–30, was heterogeneous in their demographic and clinicopathological characteristics. Compared to patients with the RS 18–25, breast cancer patients with the RS 26–30 were more likely to have aggressive tumor: larger size, higher grade, and single hormone receptor positivity. Also, we found that a 5% increased risk of overall mortality per 1 unit increase in the RS among breast cancer patients with the RS 18–30, which was consistent with a previous finding [9]. Breast cancer-specific mortality was increased by 9% per 1 unit increase in the RS among our study population with the RS 18–30.

As expected [20], chemotherapy-treated patients had more aggressive clinicopathological features. However, after adjusting for demographic and clinicopathological factors in our study, chemotherapy was significantly associated with a lower risk of breast cancer-specific and overall mortality in patients ≤ 70 years old with the 21-gene RS 26–30. This suggested that adjuvant chemotherapy might be beneficial to patients who are ≤ 70 years old and have the RS 26–30 even though they are classified as having an intermediate risk of recurrence. The study by Ibraheem et al. [9] also reported that chemotherapy was related to a 32% lower risk of overall mortality in patients with node-negative breast cancer and the 21-gene RS 26–30. In a re-analysis of the National Surgical Adjuvant Breast and Bowel Project (NSABP) B-20 trial excluding presumed HER2-positive cases, significantly better distant recurrence-free survival rates were observed in patients with the 21-gene RS ≥ 26 who received chemotherapy [4].

Our study was one of the largest studies examining the effect of chemotherapy on survival in breast cancer patients with an intermediate risk for recurrence. The SEER data which capture > 98% of incident cancer cases in 18 geographical regions provide a large number of breast cancer cases with survival outcome and the 21-gene RS [11, 21]. In addition, we selected our patient population according to the NCCN practice guidelines and focused analyses on patients with the RS 26–30, which will help decision-making for chemotherapy in patients with the RS 26–30. Our study also has several limitations mostly related to inherent limitations in the SEER database: it did not provide information on disease recurrence, endocrine therapy use, and details of chemotherapeutic regimens. Also, receipt of chemotherapy and radiation therapy was under-reported in the SEER program compared to the Medicare claims data restricted to patients aged ≥ 65 years [22]. Given that systemic chemotherapy tends to be administered to patients with better performance status which could not be assessed in this study, we cannot rule out the possibility of residual confounding by unmeasured factors in our analyses. In addition, although the distribution of the 21-gene RS did not differ between 2010 and 2015 and 2004–2009 dataset, about 5% of total breast cancer cases had HER2-positive, borderline, or undetermined status in 2010–2015 dataset just before the final exclusion step. Given that 1with HER2-positive were more likely to have the higher 21-gene RS, analyses of 2004–2009 data may have additional confounding due to lack of HER2 status. Nevertheless, when we performed sensitivity analyses including patients with HER2 positive, borderline, or unknown status in 2010–2015 data, our results did not change. Another limitation was the lack of information on menopausal status. Thus, we could not examine premenopausal and postmenopausal breast cancer, separately.

## Conclusions

Our findings suggest that adjuvant chemotherapy provides a significant survival benefit to patients with an intermediate risk for recurrence, particularly those with the 21-gene RS 26–30, and particularly for younger patients and those with high-grade tumors. Given that the 21-gene RS is a promising tool that can guide a chemotherapy decision in early-stage breast cancer patients with hormone receptor-positive and HER2-negative tumor, a shared-decision making with patients, using the multigene assay result and discussions about known risks and benefits of chemotherapy should be warranted in the era of personalized medicine.

## Additional files

Additional file 1: Figure with CONSORT diagram. Abbreviation: RS, recurrence score; SEER, Surveillance, Epidemiology, and End Results; ER, estrogen receptor; PR, progesterone receptor; HER2, human epidermal growth factor receptor 2. (DOCX 115 kb)

Additional file 2: Figure with distribution of the 21-gene RS. Blue color represents the 2004–2009 dataset, green, 2010–2015 dataset just before the final step of excluding HER2-positive, borderline, or unknown status, and red, the final analytic 2010–2015 dataset remaining only HER2-negative category after exclusion of all criteria. Percentage of the 21-gene RS is calculated in each dataset. Dotted line shows a cutoff used in this study. The mean of the 21-gene RS among datasets was not significantly different (*t*-test, *P* = 0.296 between the 2004–2009 dataset and 2010–2015 dataset before excluding HER2; *P* = 0.065 between 2004 and 2009 dataset and the final 2010–2015 dataset after excluding HER2; and *P* = 0.305 between 2010 and 2015 dataset before excluding HER2 and the final 2010–2015 dataset after excluding HER2). (DOCX 80 kb)

Additional file 3: Table with multivariate analysis for risks of breast cancer-specific and overall mortality by the 21-gene RS in sensitivity analyses that included breast cancer with HER2-positive, borderline, and unknown status in 2010–2015 (*n* = 20,943). (DOCX 15 kb) (DOCX 14 kb)

Additional file 4: Table with patients’ characteristics by chemotherapy use in patients aged ≤70 years old with the recurrence score (RS) 26–30. *Other race includes American Indian, Alaska Native, and Asian or Pacific Islander. †Other marital status includes separated, divorced, and widowed categories. Abbreviation: RS, recurrence score; No., number; ER, estrogen receptor; PR, progesterone receptor. (DOCX 18 kb)

## Data Availability

The data that support the findings of this study are available from the SEER program (https://seer.cancer.gov/), but restrictions apply to the availability of these data, which were used under permission for the current study, and so are not publicly available. Data are however available from the authors upon reasonable request and with permission of the SEER.

## Abbreviations

CI: Confidence interval
ER: Estrogen receptor
HER2: Human epidermal growth factor receptor 2
HR: Hazard ratio
ICD-O-3: International Classification of Diseases for Oncology, 3rd Edition
NCCN: National Comprehensive Cancer Network
NCDB: National Cancer Data Base
NSABP: National Surgical Adjuvant Breast and Bowel Project
PR: Progesterone receptor
RS: Recurrence score
TAILORx: Trial Assigning Individualized Options for Treatment
