# Supplementary material for: Adjuvant chemotherapy and survival among patients 70 years of age and younger with node-negative breast cancer and the 21-gene recurrence score of 26–30
Source: Breast Cancer Res. 2019 Oct 16;21:110. doi: 10.1186/s13058-019-1190-4 (PMC6796491; doi:10.1186/s13058-019-1190-4)
Supplement: Supplementary file 1 — Figure with CONSORT diagram. Abbreviation: RS, recurrence score; SEER, Surveillance, Epidemiology, and End Results; ER, estrogen receptor; PR, progesterone receptor; HER2, human epidermal growth factor receptor 2. (DOCX 115 kb) [file 13058_2019_1190_MOESM1_ESM.docx]

**Figure S1.** CONSORT diagram.


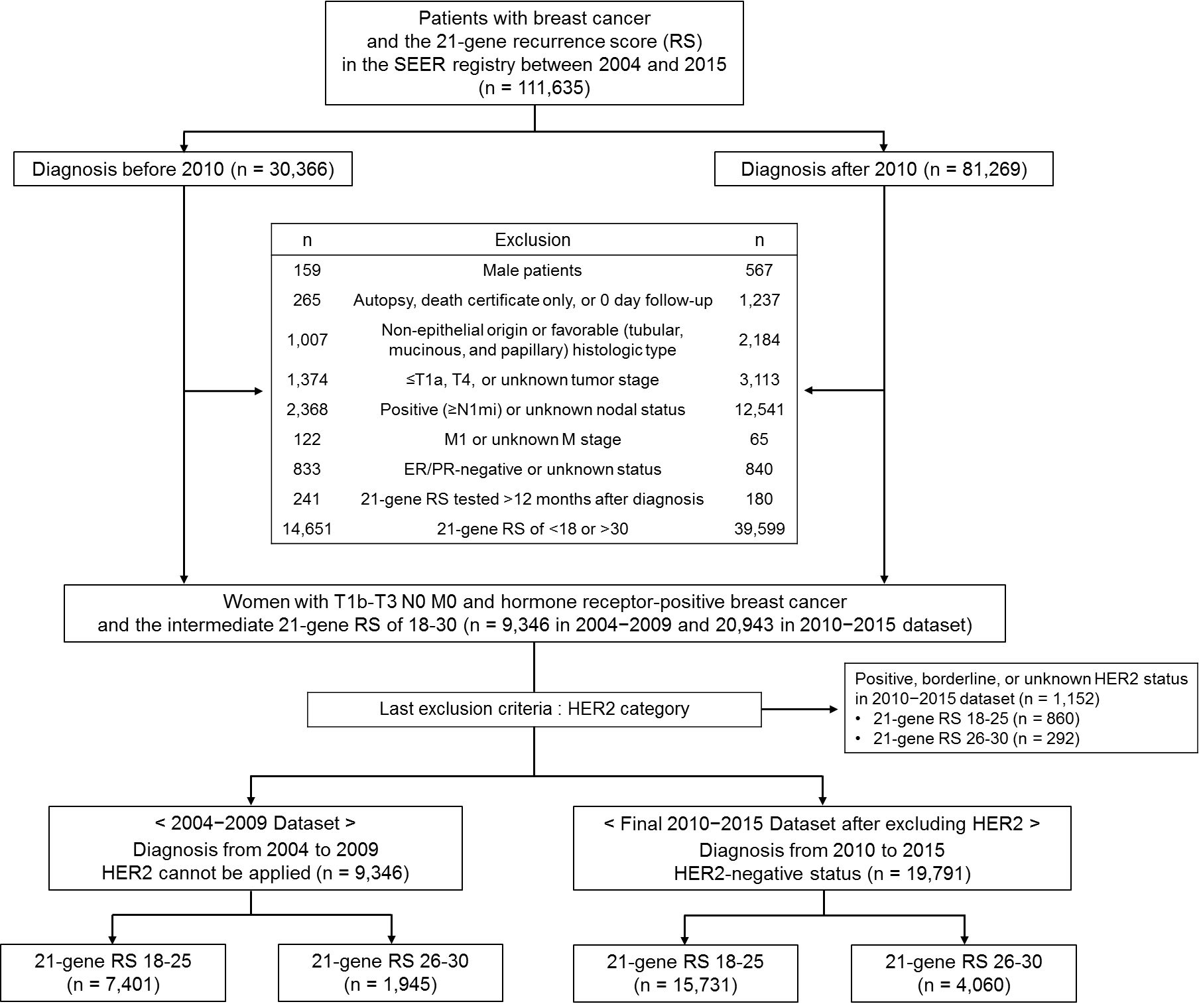


Abbreviation: RS, recurrence score; SEER, Surveillance, Epidemiology, and End Results; ER, estrogen receptor; PR, progesterone receptor; HER2, human epidermal growth factor receptor 2.
