# Supplementary material for: Adjuvant chemotherapy and survival among patients 70 years of age and younger with node-negative breast cancer and the 21-gene recurrence score of 26–30
Source: Breast Cancer Res. 2019 Oct 16;21:110. doi: 10.1186/s13058-019-1190-4 (PMC6796491; doi:10.1186/s13058-019-1190-4)
Supplement: Supplementary file 2 — Figure with distribution of the 21-gene RS. Blue color represents the 2004–2009 dataset, green, 2010–2015 dataset just before the final step of excluding HER2-positive, borderline, or unknown status, and red, the final analytic 2010–2015 dataset remaining only HER2-negative category after exclusion of all criteria. Percentage of the 21-gene RS is calculated in each dataset. Dotted line shows a cutoff used in this study. The mean of the 21-gene RS among datasets was not significantly different (t-test, P = 0.296 between the 2004–2009 dataset and 2010–2015 dataset before excluding HER2; P = 0.065 between 2004 and 2009 dataset and the final 2010–2015 dataset after excluding HER2; and P = 0.305 between 2010 and 2015 dataset before excluding HER2 and the final 2010–2015 dataset after excluding HER2). (DOCX 80 kb) [file 13058_2019_1190_MOESM2_ESM.docx]

**Figure S2.** Distribution of the 21-gene RS.


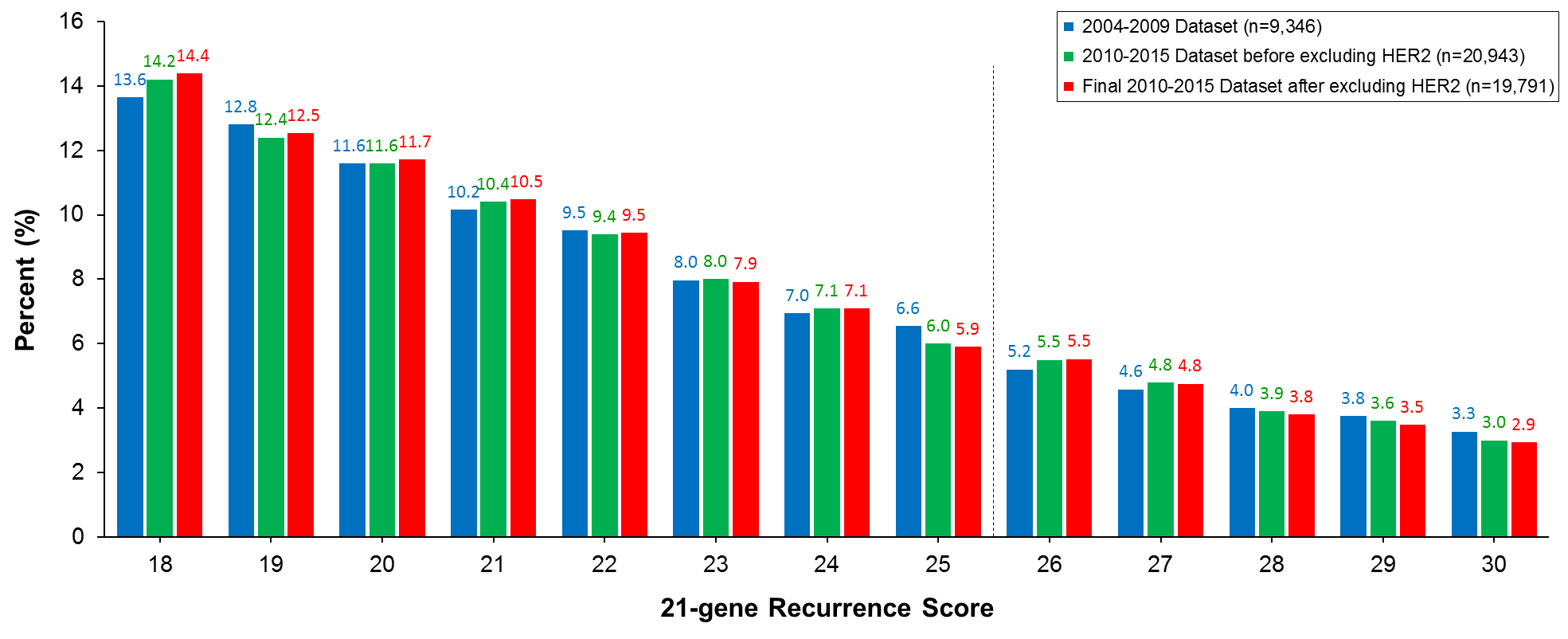


Blue color represents the 2004−2009 dataset, green, 2010−2015 dataset just before the final step of excluding HER2-positive, borderline, or unknown status, and red, the final analytic 2010−2015 dataset remaining only HER2-negative category after exclusion of all criteria. Percentage of the 21-gene RS is calculated in each dataset. Dotted line shows a cutoff used in this study. The mean of the 21-gene RS among datasets was not significantly different (*t*-test, *P* = 0.296 between the 2004−2009 dataset and 2010−2015 dataset before excluding HER2; *P* = 0.065 between 2004−2009 dataset and the final 2010−2015 dataset after excluding HER2; and *P* = 0.305 between 2010−2015 dataset before excluding HER2 and the final 2010−2015 dataset after excluding HER2).
