# Supplementary material for: Adjuvant chemotherapy and survival among patients 70 years of age and younger with node-negative breast cancer and the 21-gene recurrence score of 26–30
Source: Breast Cancer Res. 2019 Oct 16;21:110. doi: 10.1186/s13058-019-1190-4 (PMC6796491; doi:10.1186/s13058-019-1190-4)
Supplement: Supplementary file 3 — Table with multivariate analysis for risks of breast cancer-specific and overall mortality by the 21-gene RS in sensitivity analyses that included breast cancer with HER2-positive, borderline, and unknown status in 2010–2015 (n = 20,943). (DOCX 15 kb) (DOCX 14 kb) [file 13058_2019_1190_MOESM3_ESM.docx]

**Table S1** Risks of breast cancer-specific and overall mortality by the 21-gene RS in sensitivity analyses that included breast cancer with HER2-positive, borderline, and unknown status in 2010−2015 (*n* = 20,943)

|  | **Breast cancer-specific mortality** | | **Overall mortality** | |
| --- | --- | --- | --- | --- |
|  | **RS 18-25**  **(Reference)** | **RS 26-30**  **[HR (95% CI)]** | **RS 18-25**  **(Reference)** | **RS 26-30**  **[HR (95% CI)]** |
| No. of events | 88 | 51 | 292 | 116 |
| Age-adjusted model | 1.00 | 2.20 (1.56 to 3.11) | 1.00 | 1.48 (1.19 to 1.83) |
| Age and clinicopathological factors adjusted model | 1.00 | 1.77 (1.24 to 2.53) | 1.00 | 1.30 (1.04 to 1.63) |
| Age and clinicopathological and treatment factors adjusted model | 1.00 | 1.78 (1.23 to 2.58) | 1.00 | 1.31 (1.04 to 1.65) |
| HER2 as well as age and clinicopathological and treatment factors adjusted model | 1.00 | 1.78 (1.23 to 2.58) | 1.00 | 1.31 (1.04 to 1.65) |
| Continuous RS in HER2 as well as age and clinicopathological and treatment factors adjusted model* | 1.11 (1.06 to 1.17) / 1-unit RS | | 1.05 (1.02 to 1.08) / 1-unit RS | |

Abbreviation: HER2, human epidermal growth factor receptor 2; RS, recurrence score; HR, hazard ratio; CI, confidence interval; No., number.

*The 21-gene RS is entered as a continuous variable into Cox’s hazard models adjusting for HER2 status, age at diagnosis, and clinicopathological and treatment factors. HR per 1 unit increase in RS

Note: HER2 is categorized into negative, positive, and borderline/unknown status. Age at diagnosis is used by categorization into ≤50, 51-60, 61-70, and >70 years. Adjusted clinicopathological factors are year of diagnosis (2010-2011, 2012-2013, and 2014-2015), race/ethnicity (White, Black, and other), history of cancer (no and yes), marital status (married and single/other), histologic type (ductal, lobular, and mixed ductal-lobular/other), tumor stage (T1b, T1c, and T2-3), grade (I, II, III, and missing), and ER/PR status (both ER/PR-positive and either ER/PR-positive). Treatment factors are type of surgery (breast-conservation surgery and mastectomy), radiation therapy (no/unknown and yes), and chemotherapy (no/unknown and yes).
