# Supplementary material for: Adjuvant chemotherapy and survival among patients 70 years of age and younger with node-negative breast cancer and the 21-gene recurrence score of 26–30
Source: Breast Cancer Res. 2019 Oct 16;21:110. doi: 10.1186/s13058-019-1190-4 (PMC6796491; doi:10.1186/s13058-019-1190-4)
Supplement: Supplementary file 4 — Table with patients’ characteristics by chemotherapy use in patients aged ≤70 years old with the recurrence score (RS) 26–30. *Other race includes American Indian, Alaska Native, and Asian or Pacific Islander. †Other marital status includes separated, divorced, and widowed categories. Abbreviation: RS, recurrence score; No., number; ER, estrogen receptor; PR, progesterone receptor. (DOCX 18 kb) [file 13058_2019_1190_MOESM4_ESM.docx]

**Table S2** Patients’ characteristics by chemotherapy use in patients aged ≤70 years old with the recurrence score (RS) 26-30

|  | **2010−2015 dataset (*n* = 3,540)** | | | **2004−2009 dataset (*n* = 1,750)** | | |
| --- | --- | --- | --- | --- | --- | --- |
|  | **No/unknown chemotherapy**  **[*n* = 1,438; No. (%)]** | **Yes**  **chemotherapy**  **[*n* = 2,102; No. (%)]** | ***P*** | **No/unknown chemotherapy**  **[*n* = 722; No. (%)]** | **Yes**  **chemotherapy**  **[*n* = 1,028; No. (%)]** | ***P*** |
| **Year of diagnosis** |  |  |  |  |  |  |
| 2004-2005 | ─ | ─ |  | 63 (8.7) | 77 (7.5) | 0.121 |
| 2006-2007 | ─ | ─ |  | 278 (38.5) | 358 (34.8) |  |
| 2008-2009 | ─ | ─ |  | 381 (52.8) | 593 (57.7) |  |
| 2010-2011 | 458 (31.9) | 624 (29.7) | 0.159 | ─ | ─ |  |
| 2012-2013 | 478 (33.2) | 680 (32.4) |  | ─ | ─ |  |
| 2014-2015 | 502 (34.9) | 798 (38.0) |  | ─ | ─ |  |
| **Age at diagnosis (years)** |  |  |  |  |  |  |
| ≤50 | 275 (19.1) | 648 (30.8) | <0.001 | 165 (22.9) | 374 (36.4) | <0.001 |
| 51-60 | 470 (32.7) | 769 (36.6) |  | 265 (36.7) | 389 (37.8) |  |
| 61-70 | 693 (48.2) | 685 (32.6) |  | 292 (40.4) | 265 (25.8) |  |
| **Race/ethnicity** |  |  |  |  |  |  |
| White | 1,145 (79.6) | 1,664 (79.2) | 0.522 | 625 (86.6) | 868 (84.4) | 0.660 |
| Black | 146 (10.2) | 206 (9.8) |  | 45 (6.2) | 75 (7.3) |  |
| Other* | 137 (9.5) | 223 (10.6) |  | 50 (6.9) | 81 (7.9) |  |
| Missing | 10 (0.7) | 9 (0.4) |  | 2 (0.3) | 4 (0.4) |  |
| **History of cancer** |  |  |  |  |  |  |
| No | 1,255 (87.3) | 1,885 (89.7) | 0.027 | 654 (90.6) | 952 (92.6) | 0.129 |
| Yes | 183 (12.7) | 217 (10.3) |  | 68 (9.4) | 76 (7.4) |  |
| **Marital status** |  |  |  |  |  |  |
| Married | 862 (59.9) | 1,349 (64.2) | 0.001 | 467 (64.7) | 695 (67.6) | 0.606 |
| Single | 235 (16.3) | 344 (16.4) |  | 104 (14.4) | 138 (13.4) |  |
| Other† | 265 (18.4) | 333 (15.8) |  | 126 (17.5) | 166 (16.2) |  |
| Missing | 76 (5.3) | 76 (3.6) |  | 25 (3.5) | 29 (2.8) |  |
| **Histologic type** |  |  |  |  |  |  |
| Ductal | 1,151 (80.4) | 1,700 (80.9) | 0.589 | 582 (80.6) | 831 (80.8) | 0.480 |
| Lobular | 134 (9.3) | 175 (8.3) |  | 61 (8.5) | 73 (7.1) |  |
| Mixed ductal-lobular or other | 153 (10.6) | 227 (10.8) |  | 79 (10.9) | 124 (12.1) |  |
| **Tumor stage** |  |  |  |  |  |  |
| T1b | 354 (24.6) | 353 (16.8) | <0.001 | 177 (24.5) | 203 (19.8) | 0.032 |
| T1c | 729 (50.7) | 1,130 (53.8) |  | 399 (55.3) | 581 (56.5) |  |
| T2-3 | 355 (24.7) | 619 (29.5) |  | 146 (20.2) | 244 (23.7) |  |
| **Grade** |  |  |  |  |  |  |
| I | 209 (14.5) | 198 (9.4) | <0.001 | 126 (17.5) | 106 (10.3) | <0.001 |
| II | 783 (54.5) | 1,039 (49.4) |  | 385 (53.3) | 523 (50.9) |  |
| III | 426 (29.6) | 841 (40.0) |  | 191 (26.5) | 374 (36.4) |  |
| Missing | 20 (1.4) | 24 (1.1) |  | 20 (2.8) | 25 (2.4) |  |
| **ER/PR status** |  |  |  |  |  |  |
| +/+ | 1,113 (77.4) | 1,681 (80.0) | 0.065 | 529 (73.3) | 800 (77.8) | 0.028 |
| +/- or -/+ | 325 (22.6) | 421 (20.0) |  | 193 (26.7) | 228 (22.2) |  |
| **Type of surgery** |  |  |  |  |  |  |
| Breast-conservation surgery | 962 (66.9) | 1,416 (67.4) | 0.772 | 497 (68.8) | 707 (68.8) | 0.978 |
| Mastectomy | 476 (33.1) | 686 (32.6) |  | 225 (31.2) | 321 (31.2) |  |
| **Radiation therapy** |  |  |  |  |  |  |
| No or unknown | 722 (50.2) | 895 (42.6) | <0.001 | 357 (49.5) | 405 (39.4) | 0.025 |
| Yes | 716 (49.8) | 1,207 (57.4) |  | 365 (50.6) | 623 (60.6) |  |

Abbreviation: RS, recurrence score; No., number; ER, estrogen receptor; PR, progesterone receptor.

*Other race includes American Indian, Alaska Native, and Asian or Pacific Islander.

†Other marital status includes separated, divorced, and widowed categories.
